# A Novel Method for Predicting Oncogenic Types of Human Papillomavirus

**DOI:** 10.3390/diagnostics15233014

**Published:** 2025-11-27

**Authors:** Songül Çeçen Kaynak, Hilal Arslan

**Affiliations:** 1 Department of Computer Engineering, Ankara Yıldırım Beyazıt University, Ankara 06010, Türkiye; 225101412@aybu.edu.tr; 2 Department of Software Engineering, Ankara Yıldırım Beyazıt University, Ankara 06010, Türkiye

**Keywords:** high-risk HPV, TATA-box feature, CAAT-box feature, CpG island feature, machine learning

## Abstract

**Background and Objectives:** Human Papillomavirus (HPV) is a leading cause of cervical and other anogenital cancers, with over 200 known genotypes classified into high-risk, probable high-risk, and low-risk groups. While conventional diagnostic and classification approaches often rely on sequence alignment, phylogenetic relationships, or protein structure analyses, these methods are limited in scalability, cost efficiency, and generalizability to emerging HPV types. This study aims to develop a novel, machine learning-based framework for classifying HPV genotypes by oncogenic risk using genome-derived numerical features. A key objective is to introduce TATA-box, CAAT-box, and CpG-island-based features to HPV risk prediction for the first time. **Methods:** We constructed a comprehensive feature set that integrates regulatory sequence motifs (TATA-box, CAAT-box, CpG islands) with dinucleotide and trinucleotide (k-mer) composition derived from full HPV genomes. Multiple machine learning algorithms were implemented to evaluate classification performance across all risk categories. Model accuracy, precision, recall, and F1-score were calculated to assess the effectiveness and robustness of the proposed feature set. **Results:** The proposed method achieves an average precision of 0.95, a recall of 0.95, an F1-score of 0.95, and an accuracy of 97.47%. The experimental findings indicate that the proposed method not only attains high classification accuracy across all HPV risk groups but also surpasses existing models in generalizability by utilizing genomic data and novel biologically informed features. **Conclusions:** This study introduces regulatory motif-based numerical features to HPV classification for the first time and demonstrates that integrating these with k-mer descriptors yields a highly accurate and scalable machine learning model. Unlike previous studies, which often focus on specific HPV genes or a limited subset of types, our method is scalable, robust, and capable of classifying known and emerging HPV types with high reliability. This highlights its potential for real-world deployment in large-scale epidemiological screening and vaccine development programs.

## 1. Introduction

HPV is a double-stranded DNA virus that causes various cancers, primarily cervical cancer, as well as anal, vaginal, penile, head and neck cancers [1]. According to the HPV information report published in 2022 by the International Agency for Research on Cancer (IARC) and the HPV Information Centre, each year 569,847 women are diagnosed with cervical cancer and 311,365 women die from this disease [2]. Cervical cancer is ranked as the fourth-most common cancer among women worldwide. Due to its long premalignant phase, cervical cancer can be completely prevented and, if detected early, it can be treated before progressing into cancer [3].

The early diagnosis of cervical cancer is challenging because it does not present noticeable symptoms in its initial stages, making regular screenings crucial for timely detection [4]. If cervical cancer is diagnosed at a later stage, it may spread to critical organs, making recovery more challenging for patients [5]. Therefore, early detection and treatment of cervical cancer are of vital importance. The relationship between HPV infections and cervical cancer has been extensively studied in recent decades [6]. Approximately 99.7% of cervical cancer cases are associated with HPV infections [7]. Unlike other common infection-causing viruses, such as adenoviruses [8], herpes simplex virus [9], influenza viruses, and coronaviruses [10], HPV plays significant oncogenic roles in various types of cancer.

More than 220 HPV types have been identified and they are categorized into five genera based on the genetic similarity of their main capsid L1 gene: Alpha (65 genotypes), Beta (54 genotypes), Gamma (98 genotypes), Mu (4 genotypes), and Nu (1 genotype) [11]. HPV types, which exhibit genetic and oncogenic variations, are classified into three categories: high risk, probable high risk, and low risk [12,13]. The most common types of high-risk HPV in cervical cancer include HPV16, HPV18, HPV31, HPV33, HPV35, HPV45, HPV52, and HPV58, while four less common types (HPV39, HPV51, HPV56, HPV59) are also classified as high risk [12]. Most cervical, anal, vulvar, vaginal, penile, head, and neck cancers originate from these types [14,15]. Probable high risk HPV types (HPV26, HPV30, HPV34, HPV53, HPV66, HPV67, HPV68, HPV69, HPV70, HPV73, HPV82, HPV85, HPV97) are also considered potentially carcinogenic. Lastly, HPV6 and HPV11 are classified as low risk as they do not cause cancer but play a role in the development of genital warts [16]. The methods commonly used for HPV detection are based primarily on the hybridization of the PCR primer and probe, targeting the most conserved gene in the HPV genome, the L1 gene [17]. Although these PCR methods are both efficient and sensitive, they cannot detect HPV types that do not specifically bind to the designed primers and probes. An HPV genotype with a genomic sequence differing from the primer/probe designs may escape amplification and hybridization, leading to undetectability [17]. This failure in HPV detection can be easily overcome by subjecting the total nucleic acids of samples to unbiased, high-throughput sequencing, as demonstrated by Mühr et al. [11]. Gravitt et al. [18] used Oligo 5.0 software (Molecular Biologic Insights, Inc., Cascade, CO, USA) to define primer and probe sequences for HPV16 and HP18. They also tested them in laboratory settings and found the analytical specificity of HPV16 and HPV18 tests to be 100% and 97%, respectively. Primer and probe sequences for various HPV types have been identified in multiple studies in the literature [19,20,21]. Karlsen et al. [22] evaluated type-specific primers for HPV11, 16, HPV18, HPV31, HPV33, and HPV35, concluding that multiple consensus primers and type specific primers are necessary in PCR detection systems to identify all HPV carrying patients. Shin et al. [23] designed primers and probes for HPV types using evolutionary computation. However, deep learning approaches have mainly been applied to analyze Computed Tomography (CT) scans for HPV detection in head and neck cancers, rather than for designing PCR primers and probes for HPV detection [24]. The most prevalent high-risk HPV types are HPV16 and HPV18, accounting for approximately 50% and 20% of cervical cancer cases, respectively [25]. Persistent infections caused by high-risk HPV can lead to cervical cancer through precursor lesions, which develop over a long period and can be detected via cytological screening. While most of these lesions regress without treatment, detecting high-risk lesions remains a major challenge in cervical cancer screening [26]. Although various screening tests are available for early detection and treatment of cervical cancer, a lack of adequate resources for high-quality screening in developing countries makes cervical cancer the second-most common cause of cancer-related death among women worldwide [3]. Diseases associated with HPV in the cervix often lead to either under-treatment or over-treatment due to the low specificity of screening tests [27]. On the other hand, the most commonly preferred diagnostic method for cervical cancer is biopsy under colposcopy guidance, followed by staging based on clinical examination and imaging results [28]. Although this is an effective strategy, it requires a well-organized infrastructure and skilled professionals such as pathologists, cytopathologists, and colposcopy specialists [29]. Despite the implementation of many effective cervical cancer screening programs in recent years [30], the lack of sufficient healthcare infrastructure and professionals limits their application and sustainability [31,32]. As Jia et al. stated [33], HPV DNA tests could provide more objective results compared to usual liquid-based cytology. HPV tests typically focus on detecting high-risk HPV types (especially HPV16 and HPV18) that increase the risk of cervical cancer. However, studies have shown that additional high-risk types—such as HPV 31, 33, 52, and 58—also contribute significantly to cervical cancer, especially in regions like China and other parts of Asia. As a result, there is growing adoption of HPV-extended or full genotyping in cervical cancer screening programs [34]. If a widely accessible and effective HPV test kit is developed, it could be incorporated into screening programs and accelerate early detection of cervical cancer. While various tests exist for identifying HPV types, the optimal test remains uncertain. Due to these reasons, effective diagnostic tests for HPV types are needed. Drawing from a substantial body of literature, it is clear that AI techniques have significantly contributed to improving both the accuracy and capabilities of HPV diagnostic systems. A wide range of studies highlight the effectiveness of machine learning techniques in identifying risk factors associated with cervical cancer, processing biomedical data, and enhancing classification outcomes. This has been achieved through the application of various models, including decision trees, support vector machines, random forests, and deep learning architectures. Beyond HPV-specific applications, artificial intelligence has been increasingly adopted across a wide range of healthcare domains, demonstrating strong potential in disease detection, genomic medicine, and clinical decision support [35,36,37,38,39,40,41,42,43]. However, despite the promising outcomes, most existing AI-based HPV classification approaches remain limited in several key aspects. Firstly, many models rely on imbalanced, small-scale datasets such as the UCI Cervical Cancer dataset, which primarily includes demographic and clinical attributes rather than genomic features. Additionally, while several studies have used protein sequences or specific viral genes (e.g., E6/E7) for risk classification, these approaches are often type-specific and fail to generalize across the full spectrum of over 200 known HPV genotypes. Moreover, existing methods are typically trained on well-studied, high-prevalence types such as HPV16 and HPV18, offering limited predictive capacity for rare, newly discovered, or geographically distinct variants. Most importantly, many AI-based HPV models are not publicly available or scalable, and they do not effectively incorporate the full-length genomic sequences of HPV. As a result, they are constrained in their ability to predict the oncogenic potential of unknown or emerging HPV types—posing a significant gap in current diagnostic capabilities. To address these limitations, we present a machine learning-based approach that classifies HPV genotypes by oncogenic risk using features derived from the viral genome sequence. Notably, we introduce novel genomic descriptors for this task: the frequencies of core promoter motifs (the TATA-box and CAAT-box) and CpG islands, in addition to di- and trinucleotide k-mer composition. TATA-box and CAAT-box are well-known core promoter elements, and CpG islands reflect regional nucleotide composition patterns, but to our knowledge they have not been previously used as discriminative features in HPV risk classification. These biologically informed features complement k-mer statistics, providing a richer representation of HPV sequence differences across risk groups. We train and evaluate multiple ML classifiers on labeled HPV genomes. Unlike prior studies focused on single genes or a limited type, this genome-based approach scales to all HPV genera and subtypes. The high accuracy across all risk categories suggests utility in large-scale surveillance and epidemiological screening, and potentially in informing vaccine development by flagging emerging high-risk strains.

## 2. Related Works

A variety of factors are associated with an elevated risk of developing cervical cancer. Building a classification model to identify these risk factors and determine the presence of cervical cancer is both a significant and complex area of research. Various studies in the literature have used a dataset comprising 36 features that include demographic and medical records of 858 patients from the UCI (University of California, Irvine Machine Learning Repository) database. Abdoh et al. [44] identified significant risk factors for cervical cancer and proposed a classification technique based on the random forest (RF) algorithm. Due to the imbalance in their dataset, which contained fewer positive cases than negative ones, they employed the Synthetic Minority Over-Sampling Technique (SMOTE) to achieve a balanced class distribution. Their algorithm increases the number of minority class samples based on k-nearest neighbors (KNN), making class distributions more equal. Their study demonstrated that combining RF classification with SMOTE improved classification performance, achieving an accuracy of 97.6%. Dong et al. [45] used full high-risk HPV genotyping along with clinical tests in an XGBoost model to predict cervical precancer. Their model (SMART-HPV) achieved up to 98.9% AUROC for CIN2+ prediction in development, and maintained 0.78 AUROC on external cohorts. The top predictive factors included specific genotypes (HPV16, 18, 52, 39, etc.), cytology results, and number of co-infections. In another study on the same 858-patient dataset, Tanimu et al. [46] proposed a Decision Tree (DT) classification algorithm to analyze cervical cancer risk factors. Recursive Feature Elimination (RFE) and Least Absolute Shrinkage and Selection Operator (LASSO) were applied to identify the most significant attributes for cervical cancer prediction. Their proposed methods achieved 98.72% accuracy and 100% sensitivity. Chauhan et al. [47] applied various machine learning methods including XGBoost (Extreme Gradient Boosting), Naive Bayes (NB), Support Vector Machine (SVM), AdaBoost (Adaptive Boosting), DT, and KNN to predict cervical cancer, reporting the maximum accuracy of 91.17% with the XGBoost model. Similarly, Mudawi and Alazeb [48] employed ML methods such as DT, Logistic Regression (LR), SVM, KNN, RF, AdaBoost, and Gradient Boosting on the same data set, achieving 100% accuracy with RF, DT, AdaBoost, and Gradient Boosting. Mehmood et al. [5] preprocessed missing patient data using Pearson correlation, then combined RF feature selection and shallow neural networks in a hybrid approach to achieve 93.6% accuracy. Glucina et al. [49] addressed the dataset imbalance using Random Oversampling and various SMOTE variants, applying MLP, SVM, KNN, NB, and LR for classification, reaching an AUC of 0.99 using the KNN method. Alsmariy et al. [50] created a hybrid model combining DT, LR, and RF with a voting method. They used SMOTE for class imbalance and PCA to reduce non-informative dimensions, reaching 98.49% accuracy. Nithya and Illango [51] achieved 100% accuracy with C5.0 and RF, 93% with SVM, and 89% with KNN on the same dataset. Vidya and Nasira [52] proposed a hybrid ML approach for early detection of cervical cancer, using Classification and Regression Tree (CART), RF, and K-Means on a dataset from National Center for Biotechnology Information (NCBI) containing 500 records and 61 biopsy/genomic features. The CART model achieved an accuracy of 83.87%, RF reached 93.54%, and the combination of RF with K-Means achieved the highest accuracy of 96.77%.

Sahay et al. [53] used SVM and CNN algorithms to classify cervical cancer risk based on 35 risk factors from 858 patients obtained from Kaggle, achieving 86.66% accuracy with SVM and 80.0% with Convolutional Neural Network (CNN). Classifying high-risk HPV types is essential for diagnosis and treatment of cervical cancer [54]. Park et al. [55] used the textual descriptions outlining the characteristics of 72 HPV types as input documents taken from HPV Sequence Database in Los Alamos National Laboratory (LANL), and they applied DT to classify the HPV types according to their associated risk levels achieving an accuracy of approximately 81.14%. Munoz et al. [56] focused on HPV DNA detection and Polymerase Chain Reaction-based (PCR-based) typing tests. They identified HPV DNA in 90.7% of 1918 cervical cancer patients and 13.4% of 1928 control patients. Şenel [57] summarized multiple HPV ML studies and confirmed that SVM and RF were successfully applied to classify HPV genotypes with higher precision than older methods. HPV-KITE [58] is a k-mer-based pipeline (using a “Tversky index” of k-mer shingles) that rapidly genotypes HPV from Next-Generation Sequencing (NGS) data.

Ai et al. [59] analyzed the E6 region in 199 HPV16 positive DNA samples regarding the strong link between cervical cancer and HPV16/HPV18. Using Lasso, 13 amino acid mutation features were identified as discriminative for high-grade squamous intraepithelial lesions (HSIL). They built LR, RF, SVM, and KNN models, with LR achieving the best performance with an AUC score of 0.944. Kim et al. [60] used Next-Generation Sequencing (NGS) on 2436 swab samples from Korean women to identify HPV types. Their study identified that the most common high-risk HPV types were 58, 56, and 16 while the most prevalent low-risk types were 90, 54, and 81. Remita et al. [61] developed CASTOR, a virus classification platform based on ML techniques inspired by the Restriction Fragment Length Polymorphism (RFLP) method. CASTOR was tested on datasets of HPV (118 sequences), Hepatitis B virus (HBV) (230 sequences), and Human Immunodeficiency Virus type 1 (HIV-1) (597 sequences) achieving an average F1 score of approximately 0.9. Asensio-Puig et al. [62] used Genome-Wide Association Studies (GWAS) to analyze 645 HPV16 genomes, identifying 56 lineage-specific Single Nucleotide Polymorphisms (SNPs). They compared RF, SVM, and KNN models, with RF achieving 99% accuracy in classifying HPV lineages. Hao et al. [63] analyzed 48 dinucleotide (DNT) and 1536 DNT representation (DCR) features of 3485 high-quality HPV sequences and developed a deep learning model based on the E6 and E7 genes to predict oncogenic potential of selected high-quality records, a total of 2782, with complete E6 and E7 coding sequences. Their CNN model achieved 100% accuracy in identifying high-oncogenic HPVs and 95% in low-oncogenic ones. Their results indicated that the three main HPV groups (Alpha, Beta, and Gamma) were distinctly separated based on DCR characteristics within the E6 and E7 coding sequences. Karamveer and Tiwary [64] used ML techniques to predict HPV phenotypes. They conducted detailed phylogenetic and evolutionary analyses of HPV types, extracting 750 features from 422 (25 carcinogenic and 397 non-carcinogenic) HPV genome sequences. The selected 21 features derived from E2 and E6 genes achieved classification accuracies of 100% with LR, and 99.56% with SVM and KNN.

Recent protein sequence and structure-based studies have also been proposed. Kim and Eom [65] introduced a kernel-based approach using E6 protein sequences. They used a gap-spectrum kernel to calculate similarity between amino acid pairs at specific distances and applied SVM for classification. Wang et al. [66] employed SVM models to classify 72 different HPV types based on protein sequences from E1, E2, E4, E6, E7, L1, and L2 genes, reaching an accuracy of 93.15%. Joung et al. [67] applied Hidden Markov Models and SVM on 72 E6 sequences, attained an accuracy of 93.15%. Kim et al. [68] proposed an Ensemble SVM method incorporating protein secondary structures taken from LANL for HPV risk classification, achieving 95.59% accuracy.

Shetty and Shah [69] reviewed cervical cancer prediction studies from 2006–2017. SVM, KNN, RF, CART, and NN were compared, with SVM being among the most frequently used. Input data included cytological/histological data (e.g., Pap-smear images), demographic information, lifestyle habits, and medical history. The review emphasized the imbalanced nature of cervical cancer datasets and noted common preprocessing steps like k–means clustering and Gaussian filtering. While deep learning approaches show promise, their limitations include high data requirements, computational costs, and overfitting on small datasets. In another study, Rahimi et al. [70] systematically reviewed ML approaches for predicting cervical cancer patient survival. Across 13 studies, the most commonly employed models included RF (used in 6 studies), LR (4 studies), SVM (3 studies), ensemble or hybrid methods (3 studies), and deep learning techniques (3 studies). The sample sizes varied considerably, ranging from 85 to 14,946 patients. While the majority of studies utilized cross-validation, only two incorporated external validation. Reported performance metrics showed a wide range, with AUC values between 0.40 and 0.99, accuracy from 61% to 92%, sensitivity between 20% and 97%, and F1-scores ranging from 0.22 to 0.92. Although nucleotide composition-based approaches are among the most straightforward and accurate methods for characterizing novel genomes, there is still a scarcity of AI-driven research in this area. Simon et al. [71] underlined the significance of using nucleotide composition as the most basic strategy for characterizing genomes. They highlighted that dinucleotide frequencies were particularly useful for making accurate inferences when homologous relationships were insufficient. Their study demonstrated a strong correlation between sequence data and HPV types, suggesting that this positive relationship could be effectively evaluated using ML techniques for identifying high-risk HPV variants. Differentiating between carcinogenic and non-carcinogenic HPV types continues to be a significant challenge in the clinical genetic diagnosis of HPV-associated cancers. Most of the published HPV carcinogenicity prediction methods have been evaluated on only a limited number of HPV types. Therefore, there is a need for new approaches that can predict the carcinogenic potential of HPV sequences and estimate the carcinogenicity of newly identified HPV types based on their genomic composition. This study was undertaken to bridge a significant gap in the current body of scientific research.

## 3. Proposed Method

Despite the genetic diversity observed among different HPV types, members within the same risk category namely high risk, probable high risk, and low risk may share certain conserved genomic characteristics. The primary objective of the proposed method is to detect and extract these underlying similarities from the genomic data, thereby enabling a more accurate and reliable classification of HPV types based on their oncogenic potential.

**Figure 1:**
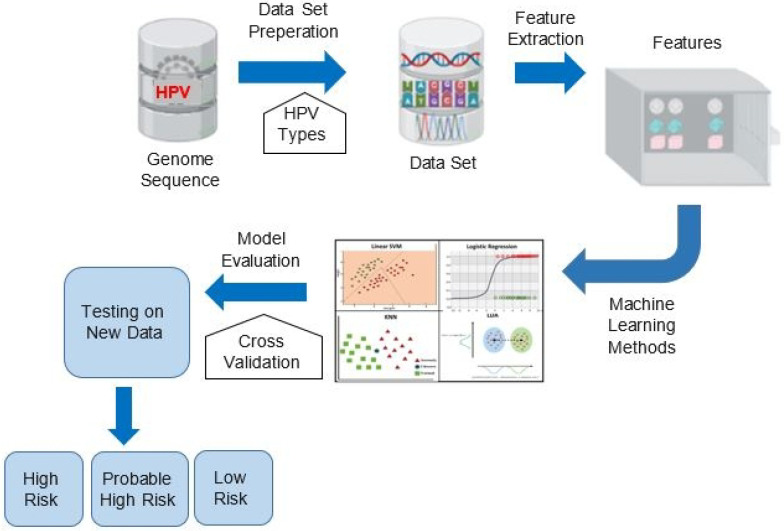
Primary stages of the proposed method.

An explanation of the methodology underlying the proposed HPV risk classification system is presented in Figure 1, and its corresponding pseudocode is detailed in Algorithm 1. The process begins with the collection of complete genome sequences from a broad range of HPV types, forming a representative and diverse dataset. This dataset serves as the input for a comprehensive feature extraction stage, where both biologically relevant and statistically informative features are derived. In the feature extraction phase, the method incorporates a diverse set of genomic descriptors. These include CpG island-related metrics as well as the occurrence ratios of the motifs, such as the TATA-box (TATAAA) and CAAT-box (CAAT). In addition, k-mer frequency distributions are computed to capture fine-grained sequence patterns that may correlate with oncogenic behavior. Notably, this study is the first to introduce CpG island-related metrics, along with the occurrence ratios of promoter motifs such as the TATA-box and CAAT-box, as numerical bioinformatics features for HPV risk classification. After the feature extraction process, multiple machine learning models were trained to capture the relationship between the derived features and HPV risk categories. Model evaluation was performed using k-fold cross-validation to ensure robustness and to prevent overfitting. Once validated, the final models were tested on previously unseen genome sequences to assess generalization peformance. Based on the classifier outputs, each test sequence was assigned to one of the three predefined risk categories: high risk, probable high risk, or low risk. This data-driven approach offers a systematic and scalable solution for HPV type risk stratification, with potential implications for early diagnosis, vaccine design, and clinical decision-making. A more detailed explanation of the feature extraction strategy and the classification step is provided in the subsequent sections.

### 3.1. Proposed Features Detecting Risk Types of HPV

One of the most critical steps in machine learning techniques is extracting meaningful and distinguishing features from the data. Accurate feature representation is fundamental to the successful training and performance enhancement of machine learning systems, as these models analyze and learn from data through these features. Extracting meaningful features that reveal genetic differences between HPV types directly affects the performance of the applied method.

In this study, distinctive features are identified from genomic sequences to differentiate HPV types based on risk level. These features have not been previously used for HPV type classification, and their introduction within the scope of this study significantly enhances its originality. The following subsections introduce new features for HPV type classification based on genomic sequence information, which has been previously applied in different areas, such as promoter region prediction [72] and COVID-19 detection [73], but not in HPV classification.
**Algorithm 1** Proposed HPV risk group prediction method     **Input:**     ●     *SeqData*: Complete genome sequences of various human HPV types     ●     *testSeq*: Sequence to be classified     **Output:** Risk classification of *testSeq* a high risk, probable high risk, or low risk      **Feature Extraction Step:**1:**for** each sequence *S* in *SeqData* **do**//  *Extraction of CpG1 and CpG2 features*2:    ratio(C) ← compute ratio of the nucleotide *C* in *S*3:    ratio(G) ← compute ratio of the nucleotide *G* in *S*4:    ratio(CG) ← compute ratio of the dinucleotide CG in *S*5:    $CpG_{1}$ = ratio(C) + ratio(G)6:    $CpG_{2}$ = ratio(CG)/(ratio(C) × ratio(G))//  *Extraction of TATA-box feature*7:    ratio(TATAAA) ← compute ratio of TATAAA in *S*//  *Extraction of CAAT-box feature*8:    ratio(CAAT) ← compute ratio of CAAT in *S*//  *Extraction of k-mer features*9:    Compute nucleotide(1-mer), dinucleotide(2-mer) and trinucleotide(3-mer) features10:**end for** **Classification Step:**11:Extract $CpG_{1}$, $CpG_{2}$, *TATA-box*, *CAAT-box*, *k-mer* features for *testSeq*12:Apply a machine learning classifier (KNN or NN is preferred)13:Output the risk group classification for *testSeq*: in high-risk, probable high-risk, or low-risk group


#### 3.1.1. CpG-Island Feature

A CpG island refers to a genomic region densely populated with cytosine and guanine nucleotides, commonly found in the genome. The CpG island feature has previously been effectively used in solving various problems, such as promoter region prediction in genomic sequences [74] and COVID-19 prediction [73]. Kottaridi et al. [75] demonstrated that in HPV16-positive women, higher methylation levels of CpG islands within the L1 gene were associated with disease severity. Yeung et al. [76] examined DNA methylation patterns and miR-23b expression in HPV16-associated cancers and found out that miR-23b was not located within a conventional CpG island. Furthermore, genomic analyses related to CpG islands have shown that high-risk HPV types tend to have fewer and smaller CpG islands compared to low-risk types [77]. In light of this information, it has been evaluated that the CpG island features described in Equations (1) and (2) have the potential to distinguish HPV types based on their risk level. This approach goes beyond the biological functions of CpG islands and aims to provide biologically meaningful input to machine learning-based classification models.

(1) $$f_{CG1}=ratio\left(“C”\right)+ratio\left(“G”\right)$$

(2) $$f_{CG2}=\frac{\mathrm{ratio}(“\mathrm{CG}”)}{\mathrm{ratio}(“C”)\mathrm{ratio}(“G”)}$$

The *ratio* function is defined as the frequency of a specified nucleotide or dinucleotide, computed by dividing its count by the total length of the sequence. Consequently, each genomic sequence is encoded as a pair of real-valued features.

#### 3.1.2. TATA-Box Feature

The TATA-box is characterized by repeating T and A base pairs (TATAAA) and is typically located approximately 25–35 base pairs upstream of the transcription start site (TSS) [78]. It has been effectively used in identifying human core promoter elements [79]. While the TATA-box has been examined in various HPV-related studies, it has not been employed as a discriminative feature for oncogenic HPV classification. Smits et al. [80] examined the transcriptional activity of HPV16 in relation to the TATAAA motif, aiming to understand how it is regulated by chromosomal changes in human cells depending on the cell type. Demeret et al. [81] demonstrated that in HPV18, the E2 protein binds to various regions, including those near the TATA-box, and suppresses the expression of cancer-related genes (E6 and E7) through cell type-specific mechanisms. Massimi et al. [82] found that the E6, E7, and E2 proteins of HPV bind to the TATA-box binding protein (TBP), which initiates gene expression in cells. This binding was shown to potentially influence the oncogenic capacity of HPV. In particular, it was observed that the E7 protein, which cannot bind to TBP, has a reduced ability to induce cancerous transformation. These findings led to the conclusion that HPV may contribute to cancer development by affecting gene regulation through the TATA-box and TBP and strong TATA signals may be present in the promoter sequences of high-risk HPV types.

While these studies underscore the functional significance of the TATA-box in HPV biology, they do not propose an approach for utilizing it as a distinguishing bioinformatic feature in classifying HPV types based on their risk group. Addressing the question of whether the TATA-box can be used to differentiate HPV types according to their risk groups, we introduced the $f_{TATA}$ feature in our study, as defined in Equation (3).

(3) $$f_{TATA}=\mathrm{ratio}\left(“TATAAA”\right)$$

where ratio(“*TATAAA*”) is calculated in a similar manner to other features, by dividing the count of occurrences of the “TATAAA” subsequence in each HPV sequence by the length of the sequence. This way, each HPV sequence is represented by a real number.

#### 3.1.3. CAAT-Box Feature

The CAAT-box is involved in transcription regulation and has a consensus sequence of GG(T/C)CAATCT [83]. Various studies on the CAAT-box and HPV have been conducted in the literature [84,85]. Desaintes et al. [86] investigated the role of the HPV16 E6 protein in the transformation process that leads to cervical cancer. Their study showed that the E6 protein could activate transcription from various viral promoters containing TATA-boxes. They also reported that mutations in regulatory elements containing CAAT-boxes and GC-rich regions reduced this activation. These findings suggest that the E6 protein acts as a “co-activator” by influencing not only the TATA-box but also regulatory elements such as the CAAT-box. Bauknecht et al. [87] demonstrated that the CAAT-box binding factor C/EBPbeta (CCAAT/enhancer-binding protein beta) could suppress viral transcription by inhibiting the binding of TBP to the TATA-box in the early promoter of HPV18. These types of interactions indicate that CAAT-like motifs can directly modulate viral promoter activity. Therefore, the frequency of CAAT motifs may serve as an indicator to help distinguish between high-risk and low-risk HPV types. For instance, Alvarez et al. [88] showed that C/EBPbeta binding sites (i.e., CAAT-binding motifs) were significantly more prevalent in the LCRs (Long Control Region) of high-risk HPV types. High-risk types such as HPV16, 18, 31, 33, and 35 contain one or more predicted C/EBPbeta binding regions in their LCRs, while such motifs are scarce in most low-risk types.

Considering the biological relevance of the CAAT-box, this study introduced a new feature, named $f_{CAAT}$, to determine whether the CAAT-box represents a statistically significant characteristic for differentiating among HPV risk groups. $f_{CAAT}$ is computed by dividing the count of occurrences of the “CAAT” substring in each HPV sequence by the total length of the respective sequence, as defined in Equation (4).

(4) $$f_{CAAT}=ratio\left(“CAAT”\right)$$

#### 3.1.4. K-Mer Feature

In the field of bioinformatics, subsequences of length k in DNA sequences are called k-mers. They are commonly used in genome analysis, sequence alignment, variant discovery, and many classification problems [73].

The k-mer feature is calculated by dividing the count of occurrences of subsequences of length k by the sequence length. For example, when k is 1, the frequency of nucleotides (A, T, C, G) is calculated and divided by the sequence length. This way, the genome sequence is represented by 4 real numbers. When k is 2, the frequency of dinucleotides (AT, AC, CG, etc.) is calculated and divided by the sequence length, resulting in 16 features for each sequence. In this study, 1-mer, 2-mer and 3-mer features are extracted, and their importance in predicting the HPV risk group is examined in detail.

Although there has been no direct study applied to HPV in this context, similar biological motifs and k-mer-based features have been shown to be effective in other virus classification tasks. For instance, the distinction between eight different virus types was achieved by using dinucleotide frequencies (CG, TT, etc.), with 100% accuracy obtained using the XGBoost classifier [89]. The VirFinder tool, which detects viral sequences, used only k-mer profiles as input without relying on any gene similarity, and this method has shown high success in comparative studies [90]. Additionally, a study on virus-host relationship prediction found that k-mer features applied to viral genomes (at the nucleotide level) carried strong discriminative signals, and the accuracy improved as the k-mer length increased [91]. These examples demonstrate that simple sequence features obtained at the motif or k-mer level can provide strong classification performance when combined with machine learning models.

### 3.2. Classification Methods

After extracting the effective features, in order to identify the risk level of the HPV genome sequences, different machine learning methods such as RF, KNN, SVM, DT, NN, and Adaptive Boosting (AdaBoost) are applied.

#### 3.2.1. Random Forest (RF)

Random Forest is an ensemble learning method that constructs numerous decision trees during training and merges their predictions to enhance accuracy and minimize overfitting [92]. Each tree is built using a randomly chosen subset of both the data and features, and the final output is obtained by combining the individual predictions—commonly through majority voting in classification or averaging in regression tasks.

#### 3.2.2. Support Vector Machine (SVM)

Support Vector Machine (SVM), first introduced by Cortes and Vapnik, is a highly effective method for addressing classification tasks [93]. Its strength lies in its ability to manage both linearly and non-linearly separable datasets, making it a widely adopted tool in the field. The fundamental concept of SVM involves identifying the optimal hyperplane that separates the data into two distinct categories. This hyperplane is selected to maximize the margin—the distance between the hyperplane and the closest data points from each class, known as support vectors [94]. A larger margin typically results in better generalization and improved predictive accuracy. In cases where linear separation is not feasible in the original feature space, SVM employs kernel functions to project the data into a higher-dimensional space, enabling the construction of a linear decision boundary. The choice of kernel function impacts the performance of SVM mostly [95,96].

#### 3.2.3. k-Nearest Neighbor (KNN)

K-Nearest Neighbors (KNN) is a supervised learning algorithm commonly applied to classification tasks. It classifies an unlabeled data point by examining the most similar examples in the training dataset, with similarity typically measured using a distance metric like Euclidean distance.

One of the defining features of KNN is that it does not create a model during training. Instead, it retains the entire training dataset and performs classification only when a new input is encountered, making it a type of lazy learning algorithm [97,98,99,100]. While this method offers flexibility, it can be computationally intensive during the prediction phase, especially with large datasets, as it requires comparing the new sample to all training instances.

#### 3.2.4. Decision Trees (DT)

A decision tree is a machine learning technique that identifies patterns in data by recursively partitioning the input space. It functions by analyzing input features and splitting the data into branches in a way that enhances the separation between output classes. Each internal node represents a decision based on a particular feature, while each branch reflects the possible outcomes of that decision. This process continues until a leaf, or terminal node, is reached. The final output of the decision tree is typically a classification label or a probability estimate that reflects the likelihood of the input belonging to a specific category.

The fundamental concept behind the decision tree approach is to break down a complex decision-making process into a series of simpler, sequential decisions [100,101,102]. The effectiveness of a decision tree classifier largely depends on the structure and quality of the tree it builds.

#### 3.2.5. Neural Networks (NN)

Neural network is a machine learning model inspired by the way the human brain processes information [97]. It consists of layers of interconnected units called artificial neurons. These layers include an input layer that receives data, one or more hidden layers that transform the information, and an output layer that produces the final result [103]. Each neuron is linked to others through connections that have associated weights and thresholds. When the combined input to a neuron surpasses its threshold, the neuron activates and sends its signal to the next layer. Neural networks improve their performance by adjusting these weights during a training phase, gradually learning to make more accurate predictions based on the data they process. The input layer consists of a neuron for each feature in the dataset, while the output layer has a single neuron that represents the final prediction.

#### 3.2.6. Adaptive Boosting (AdaBoost)

AdaBoost is a popular ensemble learning method that constructs a robust classifier by integrating several weak learners [104]. Initially, all training samples are assigned equal weights (typically 0 or 1). In subsequent iterations, the weights of the training samples are adjusted based on their classification errors, with higher weights assigned to those that are misclassified. If the error rate of a training sample is high then its weight will also be high. After each weak learner is trained, much weight is given to wrongly classified samples. By this, the following weak learner is concentrated on this mistake. A final prediction is made by joining each weak learners’ prediction with its weight. Much weight is given to weak learners who are much more successful.

## 4. Experimental Results and Discussion

This section evaluates the effectiveness of the proposed features in predicting the oncogenic potential of HPV types. A range of machine learning classifiers are utilized to assess the discriminative power of a comprehensive set of biologically relevant features, including TATA-box, CAAT-box, CpG-based attributes, as well as di-nucleotide and tri-nucleotide (k-mer) frequency representations. The integration of these features are intended to improve the classification accuracy of HPV types across clinically defined risk categories.

To ensure robust model validation and minimize the risk of overfitting, a k-fold cross-validation (k = 5) strategy is utilized. In this technique, the dataset is randomly partitioned into five equal subsets. In each iteration, four subsets are used to train the model, while the remaining subset serves as the test set. This process is repeated five times, allowing each subset to function as the test set exactly once. The experiments are conducted using MATLAB R2023b’s built-in cross-validation framework. It automatically maintains the class distribution of the high-risk, probable high-risk, and low-risk groups in each fold. In each iteration, approximately 80% of the data were used for training and 20% for testing. This procedure ensures that all samples are used for both training and testing across different folds, thereby improving the robustness and generalizability of the results. Performance metrics from these iterations are averaged to provide a reliable assessment of each classifier’s overall performance on the dataset. Before presenting the results, the experimental setup, dataset, and performance evaluation metrics employed for assessing the machine learning classifiers are detailed in the following subsections.

### 4.1. Experimental Setup

All computational experiments are performed on a system equipped with an 11th-generation Intel Core i7-1165G7 processor (2.80 GHz) and 16 GB of RAM. Feature extraction part is implemented by using the C programming language compiled and executed in a Linux environment (Ubuntu 24.04.1 LTS). The machine learning classifiers are implemented by using MATLAB R2023b. Before discussing the results, we introduce the dataset used in this study.

### 4.2. Human Genome Sequences Dataset

Genomic sequences of various HPV types are retrieved from the NCBI database [105] to construct the dataset used in this study. NCBI is a widely known bioinformatics resource that hosts comprehensive repositories such as GenBank, PubMed, and BLAST, facilitating the storage, analysis, and dissemination of genomic sequences, protein structures, biological processes, and medical information. The database includes an extensive collection of HPV-related genomic data, enabling detailed computational analysis.

According to the 2022 HPV Information Report by the International Agency for Research on Cancer (IARC) and the HPV Information Center, the classification of HPV types based on their risk levels are categorized into three groups: high-risk, probable high-risk, and low-risk as illustrated in Table 1. High-risk and probable high-risk HPV types are strongly associated with oncogenic potential and are implicated in the development of various cancers, while low-risk types predominantly result in benign lesions, such as warts. Consequently, the clinical manifestations of HPV infections are highly dependent on the specific HPV type involved.

Nucleotide sequences of the HPV types listed in Table 1 are retrieved in FASTA format from the NCBI database to construct the dataset. As of April 2025, the database contains a total of 25,791 sequences classified as high-risk, 879 as probable high-risk, and 1763 as low-risk. In this pre-processiong phase to ensure balanced representation across the classes, 2004 sequences were selected from the high-risk category, 879 from the probable high-risk category, and 1763 from the low-risk category. The sequences containing “N” have been removed. Furthermore, the descriptive headers within all FASTA files related to the sequences have been removed. The distribution of the HPV subtypes across these risk groups is detailed in Table 2, Table 3 and Table 4. For the probable high-risk and low-risk groups, all available subtypes are included due to their limited sequence numbers. In contrast, for the high-risk group, an equal number of sequences per subtype are selected to reach a total of 2004 samples, as shown in Table 2.

Although the NCBI GenBank repository collects HPV genomes from laboratories around the world, including those in Africa and Southeast Asia, the availability of whole-genome sequences is still uneven because sequencing capacity varies across countries. To address this limitation, future work will focus on adding more geographically balanced genomic datasets as they become publicly available.

This study also has some limitations. While group-level data balancing was applied, the high-risk and low-risk categories still contain subtypes that are unevenly represented. Such imbalance may affect the classifier’s ability to generalize across all HPV genotypes. Future research should therefore focus on assembling datasets with a more balanced subtype distribution and validating the model using larger, clinically diverse cohorts. Despite these limitations, the proposed framework demonstrates strong potential for integration into diagnostic and screening workflows, providing clinicians and laboratories with a reliable and cost-effective tool for HPV risk classification.

### 4.3. Performance Evaluation Measurements

In this study, the performance of the machine learning classifiers is evaluated using precision, recall, F1-score, and accuracy metrics, as formalized in Table 5 [106]. All metrics are calculated as a binary classification problem, considering each class independently.

**Figure 2:**
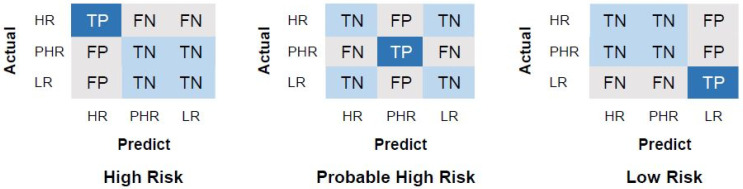
Confusion matrices for multiclasses, high risk (HR), low risk (LR), and probable high risk (PHR).

Furthermore, we represent the confusion matrices for each classifier. As an example, the computations for high-risk, probable high-risk and low-risk class are depicted in Figure 2. For the high-risk class, true positives (TP) represent the number of genome sequences correctly classified as high risk. True negatives (TN) refer to the number of genome sequences accurately predicted as non-high risk, including those in the probable high-risk and low-risk classes. False positives (FP) denote the genome sequences incorrectly classified as high risk despite belonging to the probable high-risk or low-risk classes. Lastly, false negatives (FN) are genome sequences misclassified as either probable high risk or low risk when they actually belong to the high-risk class. This evaluation approach ensures that the performance of the classifiers is comprehensively assessed across all risk categories.

### 4.4. Performance Evaluation of the Proposed CpG Feature for Predicting Risk of the HPV Types

The performance of machine learning classifiers using only CpG-based features are summarized in Table 6. Hence, the genome sequences are represented by 2 real numbers. Among the classifiers, RF exhibits the highest overall performance, achieving a mean accuracy of 92.87%, with precision, recall, and F1-score consistently outperforming other models across all classes. RF demonstrates exceptional results for the High Risk (HR) category, with a precision of 0.92, recall of 0.93, and an F1-score of 0.93. The KNN follows closely, with a mean accuracy of 89.65% and robust metrics across all categories, particularly for HR classification, where it achieves an F1-score of 0.88. The DT performs moderately well, with a mean accuracy of 85.09% and balanced metrics for HR and LR categories. However, its recall for the PHR category is comparatively lower at 0.52. The AB classifier demonstrates an average level of performance, with notable difficulty in detecting probable high-risk (PHR) types. The F1-score for both high and low-risk detection is 0.77, while the average accuracy achieves is 80.93%. The NN method shows comparatively lower performance, achieving a mean accuracy of 75.53% and an average F1-score of 0.58. Among all methods, SVM performs the worst, with a mean accuracy of 72.21%, demonstrating significant difficulty in accurately classifying the PHR category. Overall, RF stands out as the most effective classifier for CpG-based features, delivering superior results in terms of precision, recall, and F1-score across all risk categories.

**Figure 3:**
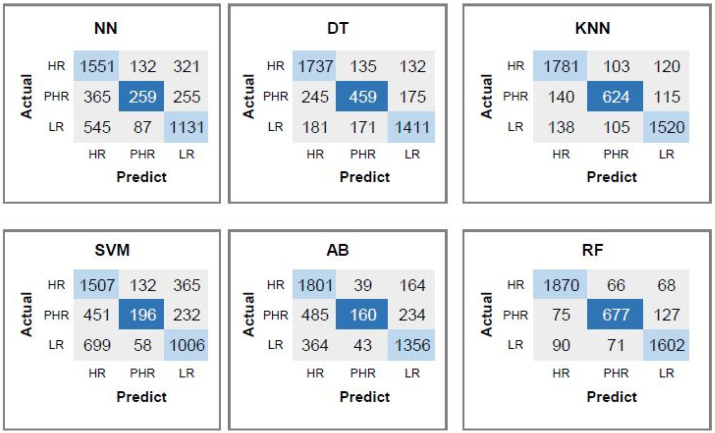
Confusion matrices corresponding to each classifier using CpG features.

As confusion matrices of each classifier presented in Figure 3 the RF classifier achieves the best performance, demonstrating high accuracy in identifying HR and LR samples, with 1870 and 1602 instances correctly classified, respectively. Misclassifications within the HR class are relatively low, with 66 instances incorrectly labeled as PHR and 68 as LR. Similarly, the LR class shows minimal confusion, with 90 samples misclassified as HR and 71 as PHR. In contrast, the PHR class exhibits a higher degree of misclassification, indicating greater difficulty in distinguishing this class from the others. Specifically, 75 PHR samples are incorrectly identified as HR and 127 as LR, while 677 are correctly classified. This suggests that the RF model struggles more with the PHR class, possibly due to feature overlap with HR and LR categories.

### 4.5. Performance Evaluation of the Proposed CpG, CAAT-Box and TATA-Box Features for Predicting Risk of the HPV Types

In this section, the integration of CpG islands, CAAT-box, and TATA-box features on the prediction of HPV type-specific risk is evaluated. Table 7 presents the performance metrics of each classifier based on features derived from CpG island, CAAT-box, and TATA-box motifs across different HPV risk categories.

The results indicate that the added features have led to overall enhancements in the performance of all classification models. Among the classifiers, the RF model stands out by attaining the highest average precision of 0.96, recall of 0.95, F1-score of 0.96, and accuracy of 96.32%. Similarly, the KNN and NN models also exhibit high classification performance with average accuracies of 95.06% and 92.24%, F1-scores of 0.91 and 0.86, respectively. These models consistently demonstrate strong predictive capability across all HPV types. SVM and DT models perform well, especially in classifying HR and LR types.

**Figure 4:**
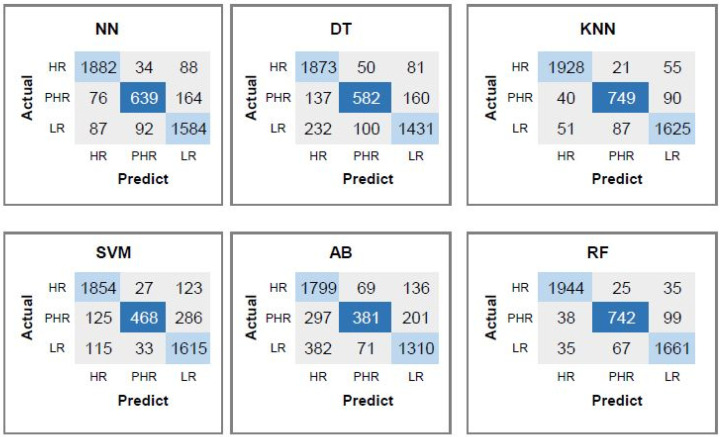
Confusion matrices corresponding to each classifier when CpG, CAAT-box, and TATA-box features are used.

Figure 4 displays the confusion matrices for each classifier, illustrating a decrease in misclassified genome sequences. Notably, the combined use of CpG features with TATA-box and CAAT-box substantially reduces the number of misclassified samples. In the HR class, the RF classifier correctly predicts 1944 instances, outperforming the KNN model, which correctly classifies 1928 samples. The RF model also results in fewer misclassifications, with 25 and 35 HR samples incorrectly labeled as PHR and LR, respectively, compared to 21 and 55 for the KNN model. This indicates that RF provides a more accurate identification of HR cases. In the PHR class, the KNN classifier shows slightly better performance, with 749 correctly classified samples, while RF correctly predicts 742. KNN misclassifies 40 PHR instances as HR and 90 as LR. In comparison, RF misclassifies 38 as HR and 99 as LR. These results suggest that the KNN algorithm is marginally more effective in distinguishing PHR samples, likely due to its sensitivity to local neighborhood structures. For the LR class, RF again achieves higher accuracy, correctly classifying 1661 samples compared to 1625 by KNN. Furthermore, RF misclassifies fewer LR samples as HR (35) and PHR (67) than KNN (51 and 87, respectively), indicating improved model precision in this class. Overall, while both models exhibit strong classification capabilities, the RF model achieves superior performance in the HR and LR categories, demonstrating better generalization and reduced error rates. Although KNN performs slightly better in the PHR class, the RF model provides a more balanced and consistent classification across all classes, making it a more robust choice for multi-class HPV risk prediction tasks.

### 4.6. Performance Evaluation of the Proposed CpG, CAAT-Box, TATA-Box, and Dinucleotide Features for Predicting Risk of the HPV Types

This section explores the contribution of the dinucleotide feature to the overall classification performance. Table 8 presents the performance of the machine learning methods. The accuracy mostly shows an increasing trend when the dinucleotid feature is added to CpG, CAAT-box and TATA-box features. Among the evaluated classifiers, the AB method shows the lowest performance, particularly for PHR types, yielding an average F1-score of 0.85 and accuracy of 92.15%. The DT classifier achieves a more balanced and robust performance across all HPV types, with an average accuracy of 93.26% and an F1-score of 0.88. SVM demonstrates high classification performance overall, with a mean accuracy of 96.79% and an F1-score of 0.94. Similarly, RF provides competitive results, reaching an average accuracy of 96.76% and an F1-score of 0.94. The NN approach also performs well, achieving an average F1-score of 0.94 and accuracy of 96.80%, demonstrating its capacity to model complex patterns within the feature set. Notably, the KNN algorithm yields the highest overall performance, especially for HR types, with nearly perfect recall (0.99) and an overall average accuracy of 97.22%, indicating its effectiveness in distinguishing between different HPV risk levels using the selected features.

**Figure 5:**
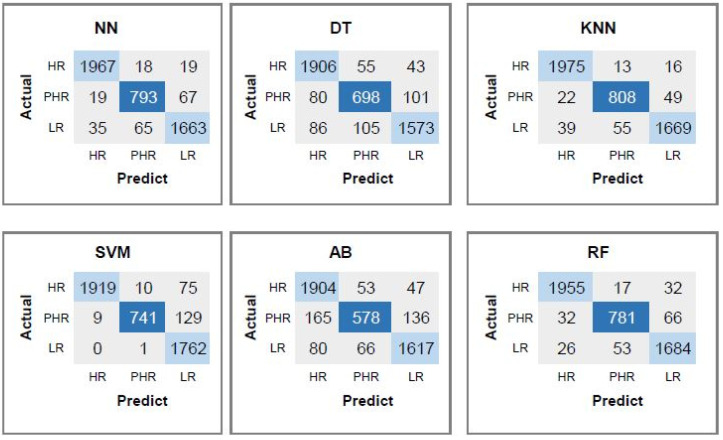
Confusion matrices corresponding to each classifier using CpG features, CAAT-box, TATA-box, and dinucleotid features.

Confusion matrices corresponding to each classifier using CpG features, CAAT-box and TATA-box and dinucleotid features are also presented in Figure 5. The KNN classifier correctly identifies 1975 HR instances, with 29 misclassifications (13 as PHR and 16 as LR). It predicts 808 PHR instances, misclassifying 71 samples.

### 4.7. Performance Evaluation of the Proposed Set of All Features for Predicting Risk of HPV Types

In this part, we analyze the performance of the machine learning classifiers when k-mer features (1-mer, 2-mer, and 3-mer) along with CpG, TATA-box, and CAAT-box features are used without applying any feature selection techniques. A total of 88 features are extracted: 4 from 1-mer, 16 from 2-mer, 64 from 3-mer, 2 from CpG, 1 from CAAT-box, and 1 from TATA-box. The results, as presented in Table 9, indicate a slight improvement in classification performance across most models compared to the previous feature set.

Based on the average performance metrics, NN attains the highest accuracy at 97.47%, accompanied by an F1-score of 0.95. Meanwhile, the KNN delivers nearly comparable results, achieving an average accuracy of 97.46%. These consistently high accuracy levels highlight the robustness and reliability of both models when applied to the enriched feature set. RF and SVM classifiers also yield consistently high performance, characterized by elevated average precision and recall values. Their respective classification accuracies are recorded as 96.87% for RF and 96.79% for SVM. DT displays a moderate to high level of performance, achieving an average accuracy of 95.05%.

**Figure 6:**
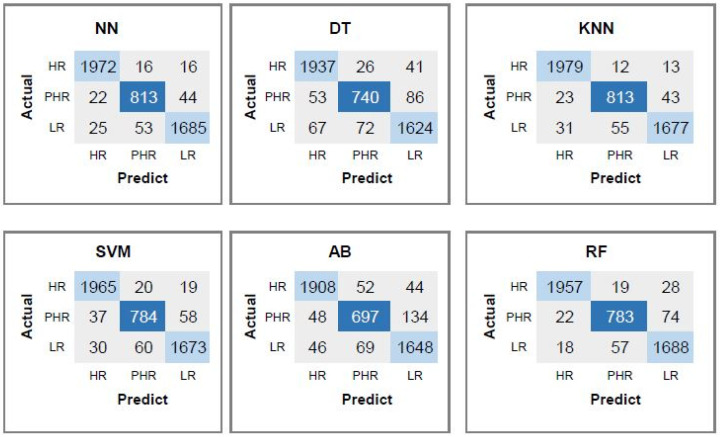
Confusion matrices corresponding to each classifier using all proposed set of features (88 features).

Confusion matrices corresponding to each classifier using all proposed set of features (88 features) are presented in Figure 6. KNN correctly classifies 1979 HR instances with 25 misclassified (12 as PHR, 13 as LR). NN correctly identifies 1972 HR instances with 32 misclassifications (16 as PHR, 16 as LR). KNN performs slightly better in HR classification, indicating higher sensitivity to high-risk individuals. Both KNN and NN correctly predict 813 PHR instances with negligible differences in how errors are distributed. For the LR category, NN yielded 1677 correct classifications and misclassified 86 samples. RF, however, performed slightly better than KNN in LR prediction, correctly classifying 1688 instances and misclassifying 75, suggesting RF’s enhanced ability to distinguish low-risk cases when leveraging the complete feature set.

Table 10 presents a comparative analysis of the mean accuracy scores obtained by various machine learning classifiers using different feature combinations. The baseline configuration employs only CpG island features, while additional models progressively incorporate CAAT-box, TATA-box, and 2-mer sequence motifs. The final column represents performance using the full set of 88 extracted features. The results present that performance consistently improves as more informative features are added. Using only CpG-related features results in relatively low accuracy across most models, with the SVM achieving the lowest performance (72.21%) and the RF performing best (92.87%) for using this minimal number of features. Among the classifiers, the RF, KNN, SVM, and NN consistently achieve higher accuracy across richer feature sets. Notably, the accuracy of KNN reaches over 97% when using CpG, CAAT-box, TATA-box, and 2-mer features. In summary, the combination of biologically relevant motifs with k-mer patterns plays a key role in achieving high classification accuracy.

The performance of the classification models was evaluated using standard metrics, including accuracy, precision, recall, and F1-score. Considering the class imbalance across HPV risk groups, macro-averaging was selected to ensure that each class contributed equally to the overall evaluation, providing a balanced view of model performance independent of class frequency. This approach was particularly appropriate given the clinical relevance of each risk group (high-risk, probable high-risk, and low-risk), where accurate prediction for each category holds distinct diagnostic and public health implications. To further validate the robustness of our findings, the results were also re-evaluated using weighted averaging, which accounts for class distribution. The classification trends and overall conclusions remained consistent across both averaging methods, confirming the reliability of the adopted evaluation approach.

### 4.8. Explainable AI for Interpreting Feature Importance

**Figure 7:**
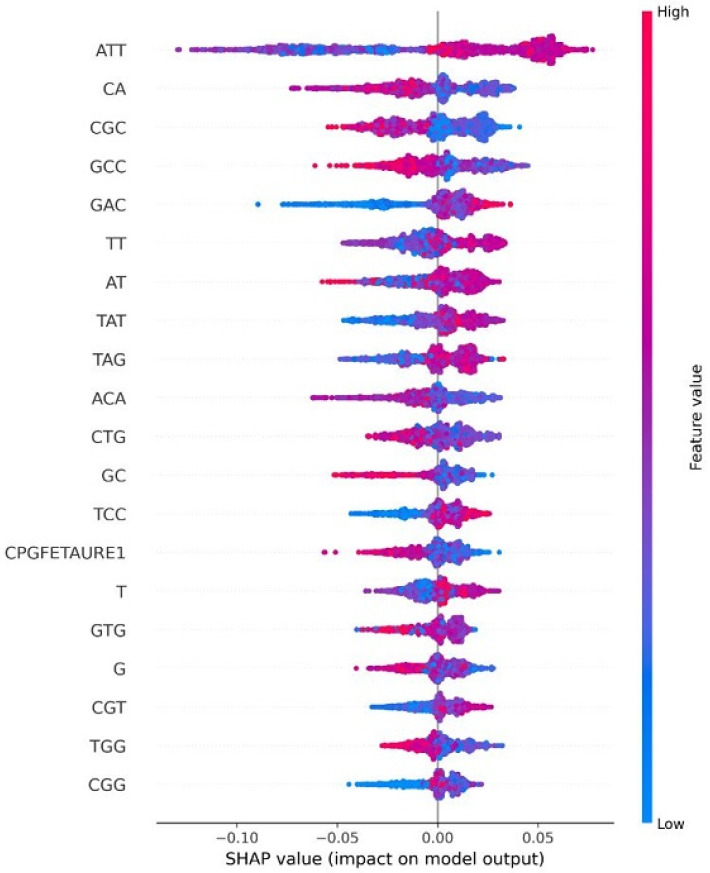
Shap value impact for High-risk HPV types.

**Figure 8:**
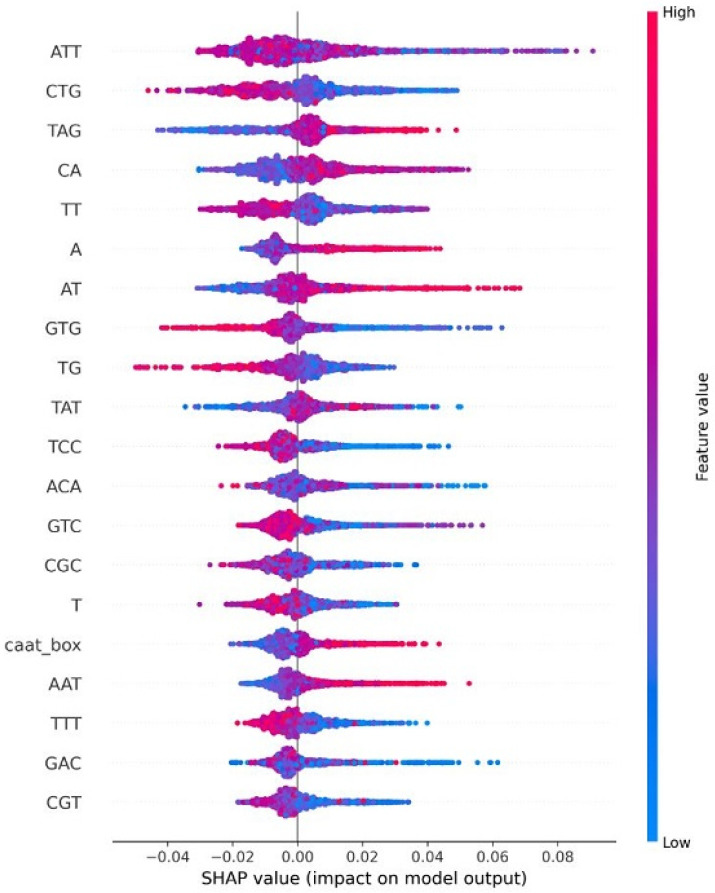
Shap value impact for Probable High-risk HPV types.

**Figure 9:**
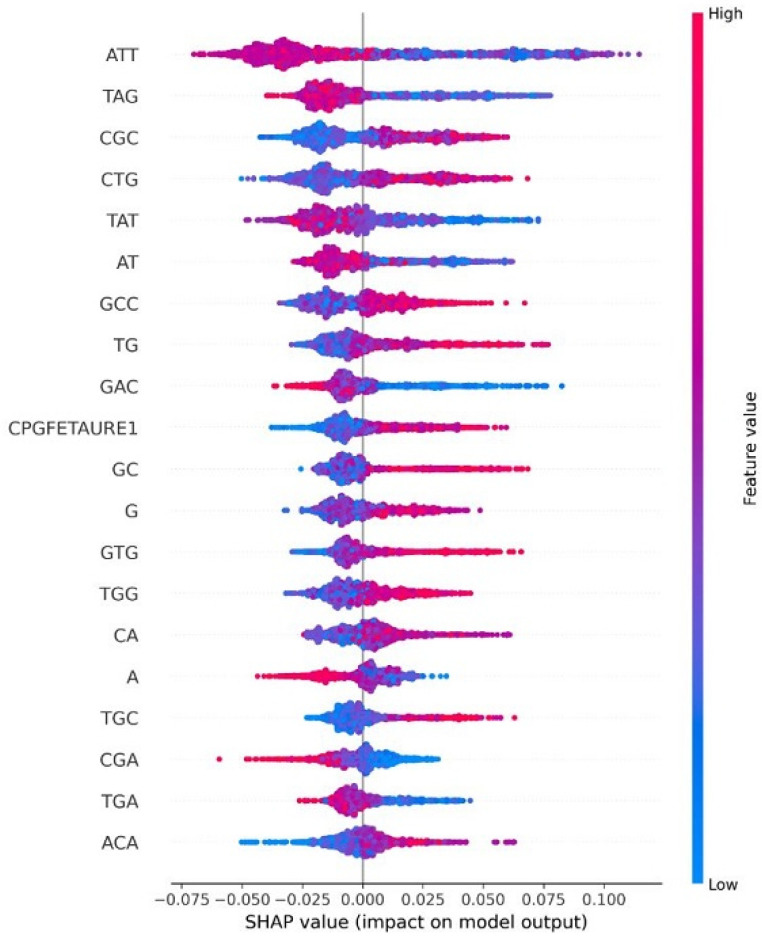
Shap value impact for Low-risk HPV types.

In this section, we explain feature importance using Shapley additive explanations (SHAP). SHAP-based feature importance analysis revealed that sequence-derived motifs play a key role in distinguishing between the three HPV risk groups. Firstly, when we analyzed the feature imprortance based on the high-risk HPV types depicted in Figure 7, the most influential features include ATT, CA, CGC, GCC, GAC, and TT, which appear at the top of the plot with the highest absolute SHAP values, indicating the strongest impact on the model’s predictions. The color gradient (blue: low value, red: high value) illustrates how feature magnitude affects risk classification. Specifically, higher values of features such as ATT, GAC, and TAT (shown in red) tend to push the prediction toward high-risk, while lower values (shown in blue) generally drive the output in the opposite direction. Notably, the CpG-related feature (CPGFEATURE1) also ranks among the most impactful variables. Its SHAP value distribution shows that reduced CpG island density significantly increases the predicted probability of a high-risk HPV type, highlighting the discriminative value of CpG patterns in this class. Secondly, for the probable high-risk group analyzed in Figure 8, ATT emerges consistently as the most influential predictor. Other important features such as CTG, TAG, CA, TT, A, AT, and GTG appear at the top of the SHAP plot, indicating strong contributions to the classification. Although the CAAT-box feature appears in the mid-to-lower region, it still contributes meaningfully, with higher CAAT-box values associated with increased predicted probability of probable high-risk HPV. Finally, for the low-risk group depicted in Figure 9, CPGFEATURE1 again stands out, but in contrast to the high-risk class, it shows predominantly negative SHAP values. This indicates that lower levels of CpG island density contribute to reduced predicted risk, suggesting a protective association in this group.

### 4.9. Comparison with Other The-State-of-the-Art Studies

This section presents a comparative evaluation of the proposed method against multiple state-of-the-art HPV classification models using the same dataset, method, and performance shown in Table 11. Ai et al. [59] investigated the E6 region in 199 HPV16-positive DNA samples and used 13 amino acid mutations as features to build a LR model for HSIL classification, achieving an AUC of 0.944 and accuracy of 84.3%.

Hao et al. [63] applied a deep CNN to HPV genomic data (2582 sequences with 1584 features) to assess the oncogenic risk of unclassified HPV types. While their approach achieved accuracy of 100% in identifying high oncogenic HPV types, the classification accuracy for low oncogenic types decreased to 95%. Wang et al. [66] employed a word-based statistical model combined with SVM to classify 72 HPV types based on their protein sequences (E1, E2, E4, E6, E7, L1, and L2), achieving an accuracy of 95.59% in identifying high-risk types. Tiwary and Karamveer [64] developed CarcinoHPVPred using logistic regression on 422 HPV genomes (750 sequence-derived features) to distinguish carcinogenic v.s. non-carcinogenic types, reporting accuracies up to 100%. In comparison, our proposed method uses only 88 biologically informed features, including motif-related attributes such as TATA-box, CAAT-box, and CpG islands, combined with k-mer frequencies. On a much larger dataset of 4646 HPV genome sequences—covering HR, PHR, and LR classes our KNN classifier achieved 98.30% accuracy for HR, 97.14% for PHR, and 96.94% for LR, with F1-scores as high as 0.98, 0.92, and 0.96, respectively. When using a feature set of only 20 features, our method still performed remarkably well, with accuracy values of 98.06% (HR), 97.01% (PHR), and 96.58% (LR) showing only a minimal decrease in performance. These results confirm that even with fewer features, the method remains highly reliable, and that the full feature set provides slight but consistent gains in classification across all risk groups. Notably, our dataset is much larger than those used in the other studies and covers three risk categories rather than a binary outcome. Even with this larger, multi-class dataset, our accuracies are comparable to or exceed those of prior models. For example, our about 98% accuracy on high-risk HPV matches or surpasses the CNN-based result of Hao et al. [63] (about 95%), and our overall performance is close to Tiwary and Karamveer’s [64] 100% result despite our use of far more samples. Moreover, our KNN model is non-parametric and does not require intensive model training like deep learning, yet it achieves similarly high accuracy. In summary, the proposed method combines simplicity, efficiency, and strong performance on a large HPV dataset, making it an effective alternative to more complex models.

## 5. Conclusions

We have developed a novel machine-learning classifier that distinguishes HPV genotypes by oncogenic risk using only genomic sequence features. A key innovation is the use of the TATA-box and CAAT-box frequencies and CpG island characteristics as input features, capturing regulatory sequence differences between HPV types based on risk level. This complements k-mer composition features and reflects known biological distinctions: for example, high-risk HPV genomes exhibit distinct CpG patterns (lower CpG observed/expected ratios) compared to low-risk types. Hence, we introduce new features for detecting oncogenic HPV types. In combination with nucleotide composition data, these features enable highly accurate classification without sequence alignment. Our classifier achieves about 97.5% overall accuracy, substantially outperforming alignment-based or protein-focused. This demonstrates that the genome composition alone can reliably separate high-risk and low-risk HPVs. The model’s alignment-free nature makes it highly scalable: it can rapidly process large numbers of full or partial-length HPV genomes or next-generation sequencing reads. Consequently, it could enhance epidemiological surveillance by enabling early identification of emerging high-risk strains. Moreover, the use of biologically interpretable features allows insight into the viral genome elements driving oncogenic potential. In future work, this framework could be extended with additional genomic signals and applied to related viral families. In future work, we will experimentally validate the model using clinical samples. Primers and probes designed from the discriminative genomic regions identified in this study are currently being developed within an ongoing funded project and will be tested across diverse clinical conditions to support eventual clinical translation. Furthermore, future research will not remain limited to current applications but will focus on developing new-generation primer and probe sequences trained on datasets containing comprehensive HPV-specific genomic sequences for analytical validation with real clinical samples. These designs aim to encompass both oncogenic and non-oncogenic HPV types. The primers and probes will be optimized to expand the limited detection capacity of existing PCR systems and cover a broader spectrum of HPV genotypes. The developed AI framework could be extended as a comprehensive model for data-rich regions and a lightweight version suitable for rapid screening in resource-constrained environments. Overall, our results demonstrate the power of integrating genomic composition and regulatory motif features in ML models to improve HPV risk prediction, with implications for screening, research, and vaccine strategy.

## Figures and Tables

**Table 1:** Classification of HPV types (Bruni et al. [2]).

HPV Risk Type	HPV Types
High Risk	16, 18, 31, 33, 35, 39, 45, 51, 52, 56, 58, 59
Probable High Risk	26, 30, 34, 53, 66, 67, 68, 69, 70, 73, 82, 85, 97
Low Risk	6, 11, 40, 42, 43, 44, 54, 55, 57, 61, 62, 64, 71, 72,
	74, 81, 83, 84, 86, 87, 89, 90, 91

**Table 2:** Total number of high risk HPV sequences in NCBI and total number of sequences used in this study.

High Risk HPV Types	Total Sequences in NCBI	Sequences Used in This Study
HPV16	12,856	167
HPV18	1609	167
HPV31	3118	167
HPV33	733	167
HPV35	1276	167
HPV39	325	167
HPV45	388	167
HPV51	476	167
HPV52	1978	167
HPV56	372	167
HPV58	2538	167
HPV59	392	167

**Table 3:** Total number of probable high risk HPV sequences in NCBI and total number of sequences used in this study.

Probable High Risk HPV Types	Total Sequences in NCBI	Sequences Used in This Study
HPV30	60	60
HPV66	379	379
HPV67	66	66
HPV68	229	229
HPV69	31	31
HPV73	45	45
HPV82	51	51
HPV85	11	11
HPV97	7	7

**Table 4:** Total number of low risk HPV sequences in NCBI and total number of sequences used in this study.

Low Risk HPV Types	Total Sequences in NCBI	Sequences Used in This Study
HPV11	794	794
HPV40	85	85
HPV42	266	266
HPV43	67	67
HPV44	107	107
HPV57	24	24
HPV62	40	40
HPV71	43	43
HPV72	16	16
HPV74	40	40
HPV81	143	143
HPV83	26	26
HPV84	22	22
HPV87	26	26
HPV86	16	16
HPV89	27	27
HPV91	21	21

**Table 5:** Performance measurements for evaluating the performance of the classifiers.

Performance Metric	Formula for Each Class *c*	Average Metric
Precision (Pre)	$\frac{TP(c)}{TP(c)+FP(c)}$	$\frac{1}{3}\sum_{i=1}^{3}Pre\left(i\right)$
Recall (Rec)	$\frac{TP(c)}{TP(c)+FN(c)}$	$\frac{1}{3}\sum_{i=1}^{3}Rec\left(i\right)$
F1-score (F1)	2 × $\frac{Pre(c)\times Rec(c)}{Prec(c)+Rec(c)}$	$\frac{1}{3}\sum_{i=1}^{3}F1\left(i\right)$
Accuracy (Acc)	$\frac{TP(c)+TN(c)}{TP(c)+FN(c)+FP(c)+TN(c)}$	$\frac{1}{3}\sum_{i=1}^{3}Acc\left(i\right)$

**Table 6:** Performance evaluation of the machine learning classifiers when CpG features are only used.

	HPV	Type Based-Results				Average Results			
Method	Type	Pre	Rec	F1	Acc	Pre	Rec	F1	Acc (%)
	HR	0.8	0.87	0.83	0.85				
DT	PHR	0.6	0.52	0.56	0.84	0.74	0.73	0.73	85.09
	LR	0.82	0.8	0.81	0.86				
	HR	0.86	0.89	0.88	0.89				
KNN	PHR	0.75	0.71	0.73	0.9	0.83	0.82	0.82	89.65
	LR	0.87	0.86	0.86	0.9				
	HR	0.57	0.75	0.65	0.65				
SVM	PHR	0.51	0.22	0.31	0.81	0.57	0.52	0.52	72.21
	LR	0.63	0.57	0.6	0.71				
	HR	0.68	0.9	0.77	0.77				
AB	PHR	0.66	0.18	0.29	0.83	0.71	0.62	0.61	80.93
	LR	0.77	0.77	0.77	0.83				
	HR	0.92	0.93	0.93	0.94				
RF	PHR	0.83	0.77	0.8	0.93	0.88	0.87	0.88	92.87
	LR	0.89	0.91	0.9	0.92				
	HR	0.63	0.77	0.69	0.71				
NN	PHR	0.54	0.29	0.38	0.82	0.61	0.57	0.58	75.53
	LR	0.66	0.64	0.65	0.74				

**Table 7:** Performance of the machine learning classifiers using CpG, CAAT-box, and TATA-box features.

	HPV	Type Based Results				Average Results			
Method	Type	Pre	Rec	F1	Acc	Pre	Rec	F1	Acc (%)
	HR	0.84	0.93	0.88	0.89				
DT	PHR	0.8	0.66	0.72	0.9	0.83	0.8	0.81	89.09
	LR	0.86	0.81	0.83	0.88				
	HR	0.95	0.96	0.96	0.96				
KNN	PHR	0.87	0.85	0.86	0.95	0.92	0.91	0.91	95.06
	LR	0.92	0.92	0.92	0.94				
	HR	0.89	0.93	0.9	0.92				
SVM	PHR	0.89	0.53	0.67	0.9	0.86	0.79	0.81	89.83
	LR	0.8	0.92	0.85	0.88				
	HR	0.73	0.9	0.8	0.81				
AB	PHR	0.73	0.43	0.54	0.86	0.75	0.69	0.71	83.41
	LR	0.8	0.74	0.77	0.83				
	HR	0.96	0.97	0.97	0.97				
RF	PHR	0.96	0.93	0.95	0.96	0.96	0.95	0.96	96.32
	LR	0.96	0.95	0.96	0.96				
	HR	0.92	0.94	0.93	0.94				
NN	PHR	0.84	0.73	0.78	0.92	0.87	0.85	0.86	92.24
	LR	0.86	0.9	0.88	0.91				

**Table 8:** Results obtained from machine learning algorithms when utilizing CpG features, CAAT-Box, TATA-Box, and dinucleotid features.

	HPV	Type Based Results				Average Results			
Method	Type	Pre	Rec	F1	Acc	Pre	Rec	F1	Acc (%)
	HR	0.92	0.95	0.94	94.32				
DT	PHR	0.81	0.79	0.80	92.66	0.88	0.88	0.88	93.26
	LR	0.92	0.89	0.90	92.79				
	HR	0.97	0.99	0.98	98.06				
KNN	PHR	0.92	0.92	0.92	97.01	0.95	0.95	0.956	97.22
	LR	0.96	0.95	0.95	96.58				
	HR	1.00	0.96	0.98	97.98				
SVM	PHR	0.99	0.84	0.91	96.79	0.96	0.93	0.94	96.79
	LR	0.90	1.00	0.95	95.59				
	HR	0.89	0.95	0.92	92.57				
AB	PHR	0.83	0.66	0.73	90.96	0.87	0.84	0.85	92.15
	LR	0.90	0.92	0.91	92.92				
	HR	0.97	0.98	0.97	97.70				
RF	PHR	0.92	0.89	0.90	96.38	0.94	0.94	0.94	96.76
	LR	0.95	0.96	0.95	96.19				
	HR	0.97	0.98	0.98	98.04				
NN	PHR	0.91	0.90	0.90	96.36	0.94	0.94	0.94	96.80
	LR	0.95	0.94	0.95	96.00				

**Table 9:** Results obtained from machine learning algorithms when utilizing 88 features.

	HPV	Type Based Results				Average Results			
Method	Type	Pre	Rec	F1	Acc	Pre	Rec	F1	Acc (%)
	HR	0.94	0.97	0.95	95.98				
DT	PHR	0.88	0.84	0.86	94.90	0.92	0.91	0.91	95.05
	LR	0.93	0.92	0.92	94.27				
	HR	0.97	0.99	0.98	98.30				
KNN	PHR	0.92	0.92	0.92	97.14	0.95	0.95	0.95	97.46
	LR	0.97	0.95	0.96	96.94				
	HR	0.97	0.98	0.97	97.72				
SVM	PHR	0.91	0.89	0.90	96.23	0.94	0.94	0.94	96.79
	LR	0.96	0.95	0.95	96.41				
	HR	0.95	0.95	0.95	95.91				
AB	PHR	0.85	0.79	0.82	93.48	0.90	0.89	0.90	94.36
	LR	0.90	0.93	0.92	93.69				
	HR	0.98	0.98	0.98	98.13				
RF	PHR	0.91	0.89	0.90	96.30	0.94	0.94	0.94	96.87
	LR	0.94	0.96	0.95	96.19				
	HR	0.98	0.98	0.98	98.30				
NN	PHR	0.92	0.92	0.92	97.09	0.95	0.95	0.95	97.47
	LR	0.97	0.96	0.96	97.03				

**Table 10:** Comparison of the performance of the proposed features based on the average accuracy values obtained from the machine learning methods.

Classifier	CpG	CpG, CAAT-Box	CpG, CAAT-Box	All 88
		TATA-Box	TATA-Box, 2-Mer	Features
DT	85.09	89.09	93.26	95.05
KNN	89.65	95.06	97.22	97.46
SVM	72.21	89.83	96.79	96.79
AB	80.93	83.41	92.15	94.36
RF	92.87	96.32	96.76	96.87
NN	75.53	92.24	96.8	97.47

**Table 11:** Comparison of the current state-of-the-art studies to classify HPV types.

**Study**	**Method**	**Number of**	**Number of**	**Acc (%)**
		**Features**	**DNA Samples**	
Ai et al. [59]	LR	13	199	84.3
**Study**	**Method**	**Number of**	**# of Genome**	**Acc (%)**
		**Features**	**Sequences**	
Hao et al. [63]	CNN	1584	2582	95
**Study**	**Method**	**# of Protein**	**Number of**	**Acc (%)**
		**Sequences**	**HPV Types**	
Wang et al. [66]	SVM	7	72	95.59
**Study**	**Method**	**Number of**	**# of Genome**	**Acc (%)**
		**Features**	**Sequences**	
Tiwary and Karamveer [64]	LR	750	25 carcinogenic	100
			397 non-carcinogenic	
**Study**	**Method**	**Number of**	**# of Genome**	**Acc (%)**
		**Features**	**Sequences**	
			2004 high-risk	98.30
Proposed Method	KNN	88	879 probable high-risk	97.14
			1763 low-risk	96.94

## Data Availability

The dataset used and analyzed during the current study is openly available at the following website: https://www.ncbi.nlm.nih.gov/labs/virus/vssi/ accessed on 4 January 2025.
